# Toxicity of Nanoscale Zero-Valent Iron to Soil Microorganisms and Related Defense Mechanisms: A Review

**DOI:** 10.3390/toxics11060514

**Published:** 2023-06-07

**Authors:** Guoming Zeng, Yu He, Fei Wang, Heng Luo, Dong Liang, Jian Wang, Jiansheng Huang, Chunyi Yu, Libo Jin, Da Sun

**Affiliations:** 1 School of Architecture and Engineering, Chongqing University of Science and Technology, Chongqing 401331, China; 2017015@cqust.edu.cn (G.Z.); 2022206022@cqust.edu.cn (Y.H.); 2021206106@cqust.edu.cn (F.W.); l632413306@163.com (D.L.); hjso419@126.com (J.H.); 2 Intelligent Construction Technology Application Service Center, Chongqing City Vocational College, Chongqing 402160, China; 3 Geological Research Institute of No. 9 Oil Production Plant of CNPC Changqing Oilfield, Yinchuan 750006, China; luoheng_cq@126.com; 4 Chongqing Yubei District Ecological Environment Monitoring Station, Chongqing 401124, China; kingj2000@163.com; 5 Department of Construction Management and Real Estate, Chongqing Jianzhu College, Chongqing 400072, China; 6 National & Local Joint Engineering Research Center for Ecological Treatment Technology of Urban Water Pollution, Zhejiang Provincial Key Laboratory for Water Environment and Marine Biological Resources Protection, Institute of Life Sciences, Biomedical Collaborative Innovation Center of Zhejiang Province, Wenzhou University, Wenzhou 325035, China; 20160121@wzu.edu.cn

**Keywords:** nanoscale zero-valent iron, biological safety, toxic effect, toxicity mechanism

## Abstract

Soil pollution is a global environmental problem. Nanoscale zero-valent iron (nZVI) as a kind of emerging remedial material is used for contaminated soil, which can quickly and effectively degrade and remove pollutants such as organic halides, nitrates and heavy metals in soil, respectively. However, nZVI and its composites can enter the soil environment in the application process, affect the physical and chemical properties of the soil, be absorbed by microorganisms and affect the growth and metabolism of microorganisms, thus affecting the ecological environment of the entire soil. Because of the potential risks of nZVI to the environment and ecosystems, this paper summarizes the current application of nZVI in the remediation of contaminated soil environments, summarizes the various factors affecting the toxic effects of nZVI particles and comprehensively analyzes the toxic effects of nZVI on microorganisms, toxic mechanisms and cell defense behaviors to provide a theoretical reference for subsequent biosafety research on nZVI.

## 1. Introduction

Nanomaterials are an important development in nanoscience and technology, and can be defined as any organic, inorganic or organometallic material that is in at least one dimension of 100 nm in three dimensions and exhibits chemical, physical and electrical properties that vary with the size and shape of the material [1]. Nanomaterials have four fundamental effects: bulk effect, surface effect, quantum size effect and macroscopic quantum tunneling effect [2]. Thus, they have some properties that are not found in conventional materials, which makes nanomaterials gain a wide range of application prospects. Recently, nanomaterials have been widely used in environmental fields [3,4], biomedicine [5], chemical engineering [6] and electronic technology [7].

Nanoscale zero-valent iron (nZVI) refers to zero-valent iron particles with a particle size of 1–100 nm, which have a large specific surface area, strong reduction, strong adsorption, and a unique oxide shell compared to bulk materials [8]. These properties have made nZVI the most widely used nanomaterial for soil and groundwater contaminated site remediation. It has been shown that nZVI can adsorb Pb^2+^, Cd^2+^, Cu^2+^ and Ni^2+^ from soil, and with the increase of nZVI concentration, the adsorption of all metals increased, and the adsorption of Pb^2+^ and Ni^2+^ reached almost 100% [9]. In addition to this, nZVI can also convert Cr^6+^ in soil into Cr^3+^, thereby reducing the toxicity to the soil [10]. However, the widespread application of nZVI can lead to the release of a large number of nZVI particles into the environment through different pathways, posing a potential risk to the local ecological environment [11,12,13]. At present, it has been shown that nanomaterials possess the ability to be ingested by organisms or inhaled into their bodies [14,15]. Microorganisms, which are key players in many basic ecosystem processes, will be the first to be exposed to the nZVI particle environment. When Yoon et al. [16] studied the toxicity of modified nZVI, they found that high concentrations of Bi-nZVI and CMC-nZVI (greater than 100 mg/L) had strong toxic effects on *Escherichia coli* and *Bacillus subtilis* in soil. Chaithawiwat et al. [17] studied the toxicity of *E. coli* after nZVI staining at 10–70 nm particle size and 1000 mg/L concentration. The results showed that the toxicity of nZVI was more serious during the exponential growth phase and decay phase of *E. coli*, and the toxic effects were strain-dependent. In general, the toxicity of nZVI was positively correlated with its concentration. However, nZVI has also been shown to exhibit stronger toxicity to Agrobacterium sp. PH-08 at low concentrations after 2 h of contact. [18]. Therefore, in order to solve the problems caused by the long-term coexistence of nZVI particles with ecosystems, it is necessary to focus on the effects of nZVI on indigenous microorganisms [19,20].

Although some studies have described the toxicological effects of nZVI on plant cells [21], animal cells [22] and microbial cells [23], few studies have been reported on the toxicological effects of nZVI on bacteria and fungi in soil. Therefore, this paper firstly outlines the achievements of nZVI in practical applications, summarizes the progress of research on the toxic effects of nZVI on microorganisms, then summarizes the mechanisms of toxicity and influencing factors in the past 10 years at home and abroad, and finally points out the problems that still exist in current nZVI toxicity research, aiming to make some references to subsequent soil microbial toxicology research on nZVI.

## 2. Application of Nano Zero-Valent Iron

With the development of urban industrialization and intensive agriculture in various countries around the world, organic and inorganic contaminants (organochlorine substitutes, heavy metals, nitrates, etc.) are spread into the soil without treatment or improper treatment. Soil pollution is insidious and long-term, and toxic and harmful substances will have a serious impact on the ecosystem after infiltrating into the soil through the material cycle [24]. A large number of studies have shown that nZVI materials, especially modified nZVI materials, have been widely used in remediation of contaminated soil due to their strong adsorption capacity, strong reducibility, low price and no secondary pollution.

### Application of Nano Zero-Valent Iron in Degradation of Soil Organic Pollutants

**Figure 1:**
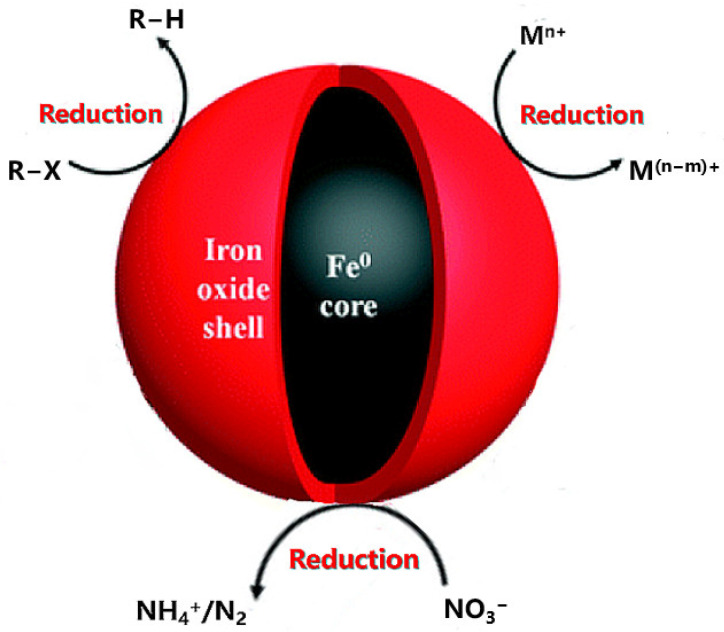
The schematic diagram of nZVI removing organic pollutants and nitrates such as organic halides. A schematic diagram of heavy metal removal by nZVI. R-X represents chlorinated organic compounds, Mn^+^ stands for inorganic/organic contaminants and NO_3_^−^ stands for metal cations [8].

Halogenated organic pollutants mainly include fluorinated, chlorinated and brominated pollutants, which are highly persistent and difficult to biodegrade, and also have toxic characteristics and can be a serious ecological hazard if they enter the environment. Stabilized nZVI has been commonly studied for the reductive dechlorination of chlorinated organic pollutants. Polychlorinated biphenyls (PCBs) are widely used in industrial production. Due to their high hydrophobicity and volatility, the natural attenuation of PCBs in soil is usually very slow and tends to accumulate in organic matter-rich soils [25,26]. Sun et al. [27] prepared montmorillonite-loaded nZVI (MMT-nZVI) and proved that it could induce a non-homogeneous Fenton reaction to degrade 2,3,4,5-tetrachlorobiphenyl (PCB67) in soil. Up to 76.38% degradation of PCB67 was achieved in an 80 min reaction with 45.99 g/kg H_2_O_2_, 29.88 g/kg MMT-nZVI and an initial pH of 3.5. DBDPE is the main material for the production of electronic products, plastics and textiles, and its unique degradability and thermal stability make DBDPE more likely to accumulate in soil [28]. Lu et al. [29] synthesized biochar-loaded nZVI (BC/nZVI) particles from bagasse at 600 °C under nitrogen protection and used them for the removal of DBDPE from soil systems. The results showed that the removal efficiency of BC/nZVI was up to 86.91% at a mass ratio of 2:1 (BC:nZVI) for 24 h of treatment. Nitrate pollution is one of the typical soil pollutants. Excessive use of nitrogen fertilizers in agriculture has led to an increase in nitrate nitrogen content in the soil, causing excessive enrichment of vegetables, and the excess nitrate entering the human body is converted to nitrite, thus increasing the risk of cancer. Zeng et al. [30] loaded nZVI on NaY zeolite and achieved nearly 100% nitrate removal after 6 h of reaction with nitrate at a dosing rate of 4 g/L, and the composite also removed more than 94% of nitrate at pH values up to 9.0. In addition, nZVI can not only degrade NO^3−^ by chemical reduction in soil remediation, but can also act as an electron donor in combination with microorganisms or coupled with bioelectrochemical system to degrade nitrate by the denitrification process [31]. The reaction process of nZVI degradation and transformation of organic pollutants and nitrates such as organic halides is shown in Figure 1. Novel contaminants in soil are not explicitly regulated by laws and regulations in terms of concentration, but are a real and present risk of environmental contamination. Chloramphenicol (CAP), a chlorinated nitroaromatic antibiotic, is an effective antibacterial drug that is widely used in a large number of developing countries due to its availability and low cost. They can pose a threat to ecosystems and human health through the production of resistant bacteria [32]. Liu et al. [33] degraded chlorinated nitroaromatic antibiotic chloramphenicol (CAP) with nZVI and showed that the degradation process can be divided into two stages: a rapid reduction of oxygen atoms in the NO_2_ group, followed by a dechlorination reaction. The degradation rate of 0.3 mM CAP in the first stage (1.8 mM of nZVI, pH 3, 6 min) was up to 97%, and the reaction resulted in a great reduction in CAP concentration.

## 3. Behavior of Nano-Zero-Valent Iron in Soil Environment

**Figure 2:**
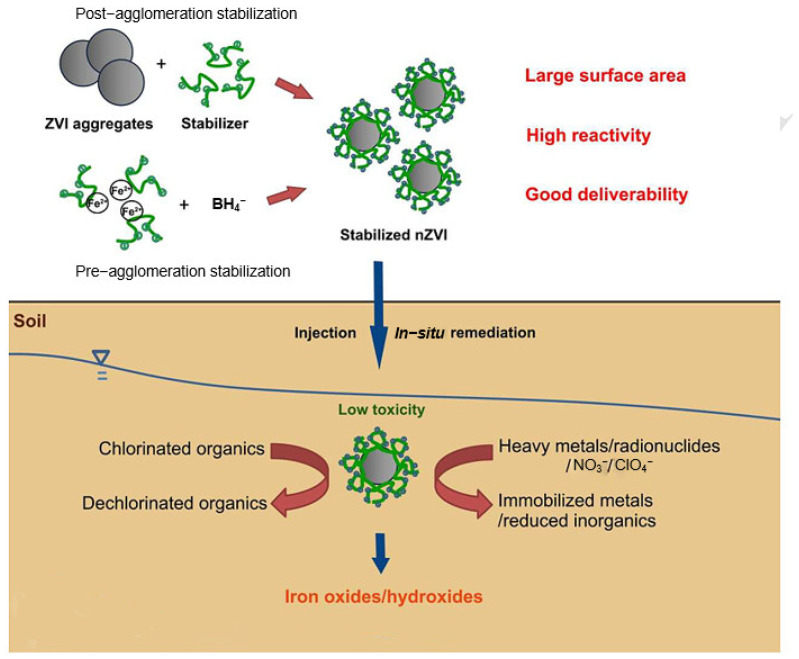
The synthesis, application, transport and fate of stable nZVI in soil [38].

Soil is an interface matrix between different substances (e.g., gas, solid, water, organic/inorganic components) and organisms, with multiple layers and complexity. The unique reaction characteristics of nZVI particles with pollutants in soil ensure the advantages of nZVI in situ remediation of contaminated soil. Figure 2 shows the schematic diagram of nZVI in situ remediation of contaminated soil. It shows the mechanism of nZVI from preparation to delivery to remediation of contaminated soil and the process of final conversion to iron oxide or hydroxide. With the wide application of nano-zero-valent iron and its composite materials in remediation of contaminated soil, more and more scholars have started to pay attention to the environmental safety and toxicological research of nano-zero-valent iron materials [34]. Soil microbial properties are also one of the indicators of soil quality impact. In practice, the toxicity of nZVI to soil microorganisms is influenced by several factors (such as the nature of nZVI, microbial tolerance, soil characteristics, etc.). Saccà et al. [35] compared the effects of nZVI on the community structure of two different types of soil and reported that in fertile soil, the number of cello flavin bacteria and Firmicutes decreased and actinomycetes increased, while the higher the sand content in the soil, the lower the number of *α-Proteobacteria* and *β-Proteobacteria*. Fajardo et al. [36] found that the most significant changes in microbial community structure were observed in nZVI-treated lead-contaminated soil. The percentage of cells belonging to the *β-Proteobacteria* subclass increased to 21.8%, while in the *ε-Proteobacteria* subclass, the percentage decreased to 6.1%. Vanzetto et al. [37] evaluated the potential toxic effects of nZVI on the growth of *Bacillus cereus* and *Pseudomonas aeruginosa* colony-forming units (CFU) in hexavalent chromium (Cr^6+^) and pentachlorophenol (PCP) nanoremediation contaminated soil. The results showed that nZVI in Cr^6+^ and PCP-contaminated soil had no toxic effect on the population of native soil bacteria. At 90 days after nZVI injection, the mean value of CFU was statistically equal, with the lowest coefficient of variation and the highest level of CFU. This is because the strains of *B. cereus* and *P. aeruginosa* are resistant to the concentrations of nZVI, Cr^6+^ and PCP, and did not cause significant disturbances in temperature, conductivity, pH and humidity over time. Furthermore, nZVI is not solely toxic to microorganisms in soil aquifers. It has been shown that nZVI inhibits the physiological activity of some microbial groups (e.g., *β-* and *γ-proteobacteria*) but promotes the growth of others (e.g., *archaea*, *LGC Gram-positive bacteria* and *α-proteobacteria*) [38,39]. The reason for this is not yet known to the academic community, but a generally accepted view is that the hydrogen produced by nZVI corrosion promotes the growth of bacteria that use hydrogen as a respiratory electron donor. Interestingly, the rapid injection of nZVI into the contaminated soil is able to promote the growth of microorganisms in the original soil to a certain extent for a period of time, and one explanation says that this is because nZVI restores some of the physicochemical properties of the contaminated soil rather than the reaction of microorganisms with nZVI [38,40,41].

## 4. Toxic Effects of nZVI on Microorganisms

**Figure 3:**
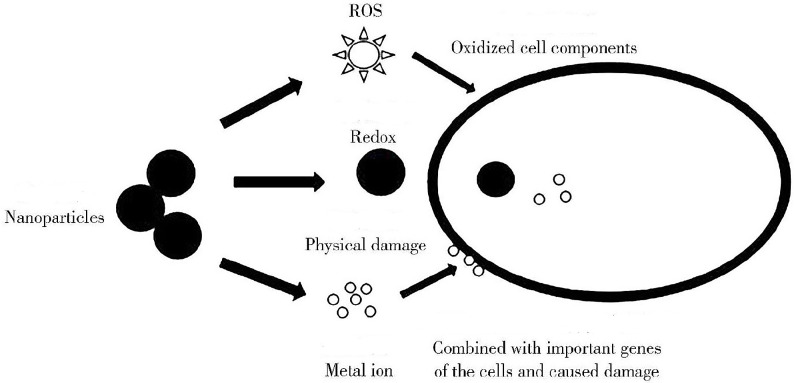
The contact diagram of nZVI and cells [48].

Microorganisms play a very important role in the ecological environment. As active components in soil, microorganisms not only regulate the organic matter, nutrient cycling and energy conversion, but also are sensitive to microenvironmental changes. In addition, their microbial activity and community structure changes are sensitive indicators of soil quality and health. Microorganisms play a key role in ecosystem function and productivity, and the toxicity of nZVI to microorganisms has received widespread attention [17,39]. As shown in Figure 3, the toxic effects of nZVI after contact with cells are complex (physical damage or release of active factors, metal ions, etc.). By studying the interaction between microorganisms and nZVI particles, we can better understand the impact of nZVI particles on the ecological environment. In the past decade, more and more studies have evaluated the toxicity of nZVI to microbial species (see Table 1).

The size and dose of nZVI have a significant effect on its toxicity. Generally, the particle size decreases and the toxicity increases. For example, the toxicity of nano-zero-valent iron is greater than that of micro-zero-valent iron [40]. However, when the particle size is less than 50 nm, the bactericidal effect of nZVI on Gram-negative bacteria is not obvious, indicating that as the particle size of nZVI further decreases, the agglomeration phenomenon is serious, which reduces its bactericidal activity [41]. The sensitivity of bacteria to the toxicity of nZVI is different in different growth cycles. The bacteria in the slow and stable phases are more resistant to the toxicity of nZVI, while the bacteria in the logarithmic growth phase and the decline phase can be rapidly inactivated by nZVI [42]. Environmental factors such as oxygen, pH and ionic strength can affect the toxicity of nZVI. For example, nZVI is significantly more toxic to *E. coli* under anaerobic conditions than under aerobic conditions [43,44]. nZVI leads to changes in bacterial community structure such as bacterial species and colony number composition, which in turn affects chemical reactions and microbial ecological effects in soil and groundwater environments [45]. The toxicity of nZVI to Gram-positive bacteria and Gram-negative bacteria is different. Studies have shown that Gram-positive bacteria are significantly resistant to nZVI toxicity due to thicker cell walls and tight cross-linking of peptidoglycan, and in some cases nZVI can even promote the growth of Gram-positive bacteria such as *Bacillus subtilis*, while nZVI has a good inactivation effect on Gram-negative bacteria such as *Escherichia coli* [46]. The surface modification of other less active metals on nZVI has a significant effect on bacterial toxicity. For example, after the surface modification of copper on nZVI to form Fe-Cu bimetallic nanoparticles, the surface Cu particles improve the efficiency of electron transfer from the iron core to the outside through the electrochemical corrosion mechanism, resulting in stronger bactericidal activity than nano-iron and nano-copper [47]. Table 2 demonstrates the toxic effects of nZVI on microorganisms.

**Table 1:** Toxic effects of nZVI on different microorganisms.

Species of Bacteria	Characteristics of nZVI	Toxic Effect	Reference
*Escherichia coli*	10–70 mm 1000 mg·L^−1^	The growth of *E.coli* was inhibited by nZVI with a particle size of 10–70 mm and a concentration of 1000 mg/L, and the toxicity of *E. coli* was more serious during the exponential growth phase and the decline growth phase.	[17]
	nZVI-B (precipitated with borohydride); size: 20–100 nm. Concentration: 1000 mg·L^−1^ nZVI-T (produced by gas phase reduction of iron oxide under H_2_); size: <100 nm Concentration: 4000 mg·L^−1^	The significant negative effects on bacteria were only found in the first two leachates. The highest nZVI toxicity to *E. coli* was detected in the aqueous phase of the slurry treated with nZVI-B. nZVI-T did not show a negative impact.	[49]
*Pseudomonas putida*	10–70 mm 1000 mg·L^−1^	nZVI showed more serious toxicity to the exponential phase and the decline phase of *Pseudomonas putida*.	[17]
	50–100 nm 10 μg/L–1.0 g/L	When *P. putida* cells were exposed to nZVI at concentrations as low as 100 μg/L, they died significantly. Negative reactions could not be detected after the nanoparticles were continuously exposed to oxygen.	[50]
*Bacillus cereus*	<50 nm 150 g/kg	No toxic effect was detected 90 days after injection of nZVI.	[37]
*Pseudomona saeruginosa*	50 g/kg	No toxic effect was detected 90 days after injection of nZVI.	[37]
*Klebsiella oxytoca*	80–120 nm 1000, 5000 and 10,000 mg·L^−1^	There are some bacterial cells around, but no obvious morphological changes. It adhered to the cell surface without obvious cell damage.	[51]

**Table 2:** Harmful effects of nZVI on microorganisms.

Properties of nZVI	Microbial Properties	Influence	References
Particle size	Gram−	When <50 nm, the toxicity decreased.	[41]
	Gram+	The smaller the particle size, the stronger the toxicity.	[40]
Dosage	Gram−	The duration of low concentration toxicity effect was stronger than that of high concentration.	[52]
	Gram+		
Aging	Gram−	The toxicity decreased significantly after oxidation.	[42,43,53,54]
	Gram+		
Modification method	Gram−	CMC/nZVI effectively alleviated the damage of cell membranes.	[55]
	Gram+	Negatively charged polymer coatings reduce toxicity	[56]
		The toxicity was significantly enhanced after modification of active metals.	[47]

## 5. Factors Affecting the Microbial Toxicity of nZVI

### 5.1. Properties of nZVI

In general, the toxic effects of nZVI on bacterial species tend to increase with the increase in nZVI concentration [18]. However, a study showed that the toxicity of 10,000 mg/l nZVI to *B. cereus* was less than 1000 mg/L after 2 h, and significant toxicity was still observed after 24 h, but only at the lowest concentration [52]. This is related to the properties of nZVI itself, because the aggregation speed of nZVI particles increases with increasing concentration, and the aggregation of large particles will settle down from the suspension, resulting in the decrease of nZVI toxicity, while the aggregation of nZVI at low concentration is relatively weak and can maintain toxicity for a long time. An et al. [54] found that the growth of *Pseudomonas stutzeri* was inhibited at the initial stage of contact with nZVI, and then the growth gradually returned to normal. Similarly, highly oxidized (mature) nanoscale zero-valent iron particles were less harmful to the microbial population compared to fresh nZVI [42]. Saccà et al. [57] confirmed that the toxicity of nZVI gradually decreased after 10 min of contact between *P. stutzeri* and nZVI. It may be due to the fact that nZVI polymerizes and settles or gradually oxidizes with time, thus reducing nZVI toxicity. Interestingly, Gonzalo et al. [58] found a colloidal singularity for nZVI aggregates at 0.1–0.5 mg/L (a phenomenon where lower particle size nZVI exhibited higher surface charge and total surface area when suspended in the experiment), and in this narrow low concentration range, nZVI aggregates instead exhibited better stability. In the nZVI-algae suspension system, nZVI at the colloidal singularity did not exhibit toxicity to microalgae.

### 5.2. Surface Modification and Soil Properties of nZVI

**Figure 4:**
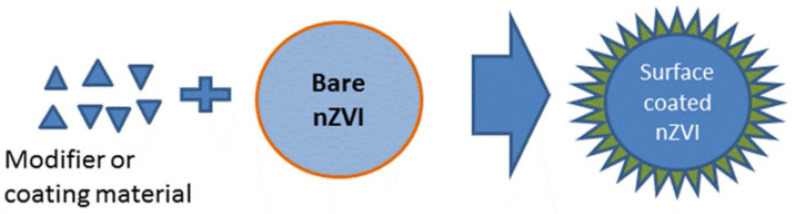
Surface coating nZVI after using modifier [60].

**Figure 5:**
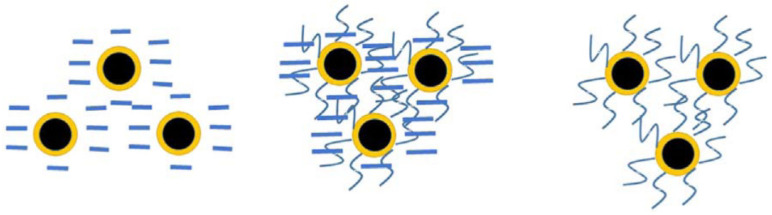
Possible indicative stabilization mechanisms of surface coatings [61].

Figure 4 shows both positive and negative in terms of the toxic effects of surface modification on nZVI. Due to the high activity of nZVI and the magnetic attraction between its particles, nZVI is prone to agglomeration and oxidation, which affects the efficiency of treating pollutants, and nZVI is generally modified when used. The spatial repulsion provided by the negatively charged polymer coating can reduce the adhesion between the bacterial cell wall and nZVI, thereby reducing the toxicity of nZVI particles [56]. Surface modification of nZVI can increase stability (Figure 5). nZVI is typically modified by CMC, a typical polyelectrolyte, which is commonly used as a stabilizer to modify nZVI and can reduce damage to cell membrane integrity or decrease oxidative stress responses. This may be due to CMC acting as a hydroxyl radical scavenger, preventing cells from oxidative stress [55]. In addition, the modification of nZVI by other metals (e.g., Ni, Ag) increases its toxicity. It is reported that Ni may lead to decreased cell growth. For Fe/Ag nanoparticles, each of them has at least some degree of antibacterial activity. Soil organic matter has also been shown to reduce the toxicity of nZVI. Pawlett et al. [59] investigated the effects of nZVI on the microbial biomass of arbuscular mycorrhizal fungi and gram-negative bacteria in three different types of soil. The results showed that the inhibitory effect of nZVI on microorganisms directly depends on the soil, which has more organic matter content. Therefore, the type of stabilizer and the presence of organic matter are also factors to be considered when assessing the toxicity of nZVI.

## 6. The Toxic Mechanism of nZVI on Microorganisms

**Figure 6:**
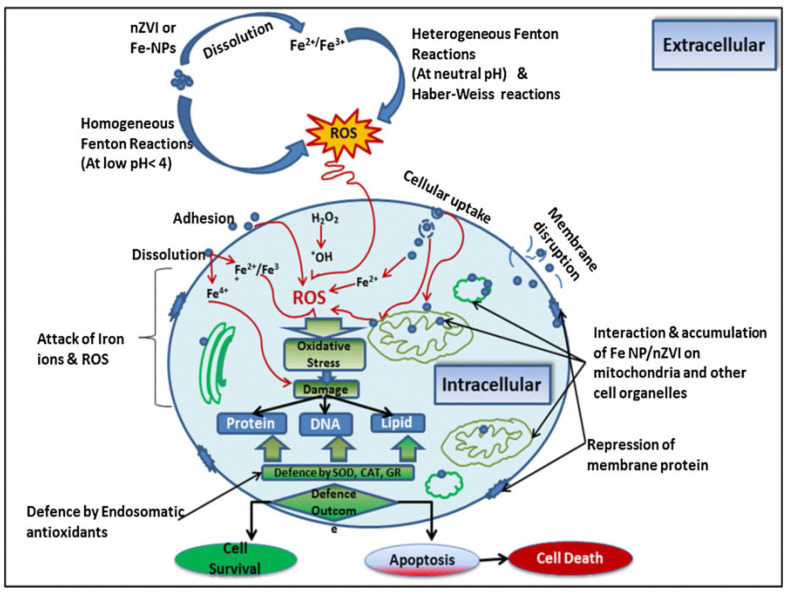
Possible toxicity mechanism and antagonism between nZVI and biological cells [60].

In the past decade, while the toxic effects of nZVI on microorganisms have been revealed, there have been many studies on the mechanisms of its toxicity, but no uniform conclusion has been reached. According to the literature, the possible mechanisms of nZVI toxicity include the following four types: morphological or functional damage of microbial cell membranes, oxidative damage, release of iron ions and genetic damage (see Figure 6).

### 6.1. Microbial Cell Membrane Morphology or Function Damage

nZVI can simply adhere to microorganisms by virtue of its physicochemical properties, such as Brownian motion, electrostatic attraction, Coulomb attraction, hydrogen bonding and even dipole-dipole collaboration. The adsorption of nZVI on microbial cells leads to direct contact with cell membranes. Lv [62] found that the inactivation of bacteria is closely related to the damage of cell membranes by studying the interfacial reaction between nZVI and cell membranes. By reacting with amino, carboxyl and ester on the membrane surface, nZVI leads to the decrease of membrane protein and polysaccharide components, thus destroying the cell membrane structure. The nZVI particles and their oxides may also block the ion channels on the cell membrane, resulting in the collapse of cell membrane potential and loss of selective permeability.

### 6.2. Oxidative Damage

Reactive oxygen species generation and oxidative stress reactions are currently recognized by academia as the main ways in which nanomaterials cause biotoxicity [63]. Due to the small size of nanomaterials, large specific surface area and many electron donor and acceptor activity sites on the particle surface, they can easily interact with molecular oxygen (O_2_) to form reactive oxygen species (ROS), which mainly include O_2_, O^2−^, H_2_O_2_ and ·OH. This type of excessive reactive oxygen species cannot be removed in time by the intracellular antioxidant defense system, so they can accumulate in large amounts in cells, resulting in an imbalance between intracellular oxidation and antioxidant status. This results in oxidative stress, which causes macromolecules such as lipids, proteins and nucleic acids to denature, damaging cell structure and eventually causing cell death. [56] At the same time, nZVI can also reduce the activity of superoxide dismutase (SOD) in cells, and SOD is a specific enzyme that can maintain the balance of cell oxidation and antioxidant system. The decrease in its activity indicates the increase of free radicals in cells, which will further aggravate oxidative damage [22]. Krittanut et al. [64] exposed wild *E. coli* strains and *E. coli* mutant strains lacking antioxidant genes to nZVI, respectively. The results showed that mutant strains were more vulnerable to damage, which confirmed that oxidative damage was one of the toxic mechanisms of nZVI.

### 6.3. Release of Iron Ions

At the initial stage of cell exposure to nZVI, nZVI is rapidly oxidized, resulting in nZVI with Fe^0^ as the core being wrapped by iron oxide in the outer layer. Once exposed to the external environment, the thickness of the external oxide layer of the nanoparticles increases rapidly [65]. Fe^2+^ and Fe^3+^ are continuously released. Trace amounts of Fe ions are indispensable for the growth of living organisms, but when there is an excess of Fe ions, it can be harmful to the organism. The reaction of releasing iron ions until the zero-valent iron core is completely oxidized, and the relevant equation is as follows [66]:

$${\mathrm{Fe}}^{0}+2H_{2}O\to {\mathrm{Fe}}^{2+}+H_{2}+2{\mathrm{OH}}^{-}$$

$${2\mathrm{Fe}}^{0}+2H_{2}O+O_{2}\to 2{\mathrm{Fe}}^{2+}+4{\mathrm{OH}}^{-}$$

$$4{\mathrm{Fe}}^{2+}+{4H}^{+}+O_{2}\to {4\mathrm{Fe}}^{3+}+4H_{2}O$$

Normally, Fe^2+^ is preferentially released on the surface of nZVI relative to Fe^3+^, the former being released on the surface of nZVI. In contrast, the strong reducing nature of nZVI decreases the permeability of the cell membrane and facilitates the entry of iron ions into the cell to react with H_2_O_2_ to generate excess ROS, causing oxidative stress and subsequent cell death [67]. The equations for the involvement of Fe^2+^ and Fe^3+^ in the intracellular Fenton reaction are as follows:

$${\mathrm{Fe}}^{2+}+H_{2}O_{2}\to {\mathrm{Fe}}^{3+}+\mathrm{OH}\cdot +{\mathrm{OH}}^{-}$$

$${2\mathrm{Fe}}^{0}+{3H}_{2}O_{2}\to {\mathrm{Fe}}^{2+}+\mathrm{OO}H^{-}+H^{+}\to {\mathrm{Fe}}^{2+}+2H^{+}+2O_{2}^{-}$$

$${\mathrm{Fe}}^{2+}+H_{2}O_{2}\to {\mathrm{FeO}}^{2+}+H_{2}O$$

Fe^2+^ and Fe^3+^ are inevitably and gradually transformed into iron oxides in the environment. Qiu et al. [68] exposed nZVI to luminescent bacteria for 36 d and observed that nZVI was no longer even toxic to the luminescent bacteria, suggesting that the toxicity of nZVI decreases with time. It was confirmed that the release of ions at the initial stage was one of the reasons for the toxicity exhibited by nZVI, but the subsequent continuous transformation of iron ions led to a gradual decrease in its toxicity.

### 6.4. Genetic Damage

With the increasing application of nanomaterials in the engineering field, there is also a growing interest in the impact of nZVI at the genetic level. Gomes et al. [69] examined the effect of nanoparticles and ions on blood cell genotoxicity by the comet assay and showed that exposure to both copper and silver in nanoparticle and ion forms induced DNA damage in blood cells, but the ion form had higher genotoxicity than the nanoparticle form. Neenu et al. [70] found that the genetic effects caused by nanomaterials include chromosome division, DNA strand breaks, point mutations and changes in gene expression profiles. Neenu further pointed out that nanomaterials can also cause carcinogenesis in the organism and even aberrations in the genes of offspring. The genetic damage caused by nanomaterials needs to be discussed at the molecular level, and this molecular toxicity is chronic and difficult to observe. To fully assess the genetic damage caused by nanomaterials to organisms in the environment, long-term observations of organisms exposed to nanomaterials are required, but there is currently little data in this regard, and scholars have not developed a uniform understanding of the genetic damage caused by nZVI, so it is also difficult to determine. The magnitude of the effects of nZVI at the genetic level is still to be determined and will need to be studied.

In fact, the toxicogenic mechanism of nZVI may not be unique, but may be a combination of multiple mechanisms. In addition to the four points mentioned above, the mechanism of toxicity may also be that nZVI has strong adsorption properties and will actively adsorb toxic and harmful substances in the environment, such as thr heavy metals As^3+^ (metalloid), Hg^2+^ and Pb^2+^, and further exhibit toxicity in concert with these heavy metals [71,72]. In order to determine the toxicity mechanism, it is also necessary to consider the environmental factors and the nature of nZVI itself.

## 7. Cell Defense Behavior

**Figure 7:**
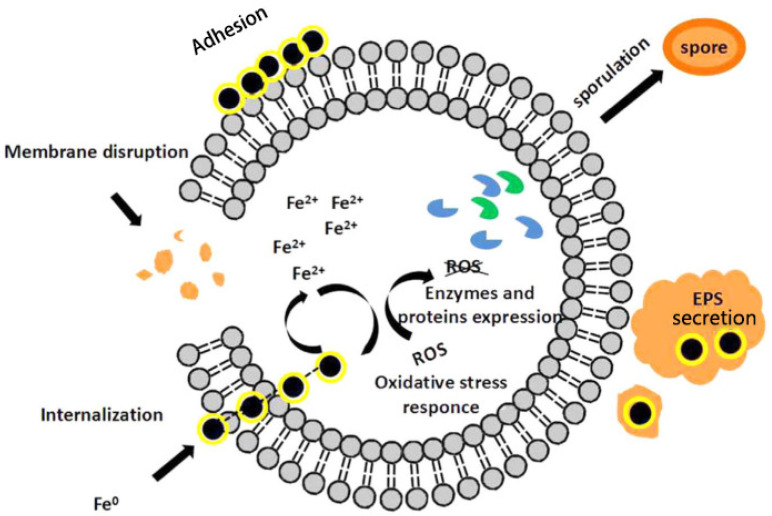
The proposed reaction mechanism of microbial toxicity when exposed to nZVI [61].

**Figure 8:**
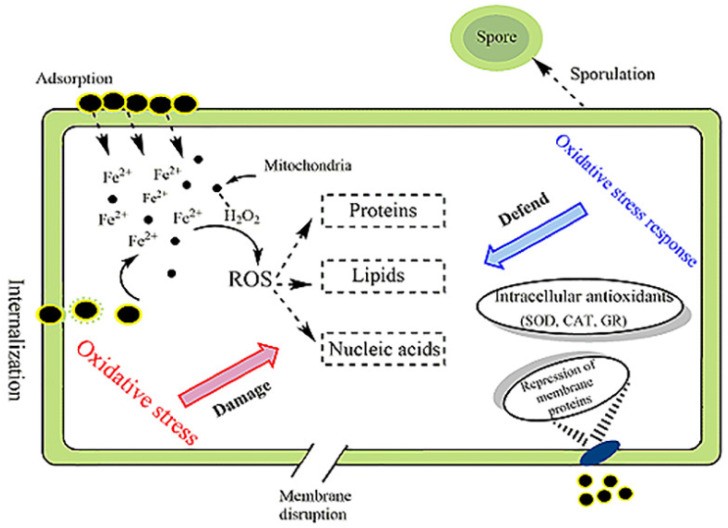
A simplified picture of the potential toxicity mechanism and cellular defense behavior of nZVI particles [34].

The mechanistic response of bacterial or fungal cells to resistance induced by nZVI toxicity is being elucidated. It has been shown in the literature that changes in protein expression indicate that cells trigger a molecular response to counteract the effects of nZVI through two main mechanisms: inhibition of iron uptake by membrane proteins; and overproduction of proteins to scavenge ROS, thereby reducing intracellular oxidative stress [56]. Saccà et al. [73] demonstrated that some membrane proteins and transporters were down-regulated after exposure to nZVI in *P. stutzeri*, which plays an important role in nutrition and iron absorption, and that a decrease in protein abundance can reduce iron availability intracellularly and cause intracellular oxidative damage. A unique defense measure was found in *Bacillus cereus*, a common Gram-positive bacterium in soil. When *Bacillus cereus* is exposed to nZVI particles, asymmetric septa are formed in the cell, which are typically in the early spore-forming phase, so that it can survive the stressful environment and regulate the expression rate of specific proteins [52]. According to the study of Saccà et al. [51], *K. oxytoca* may establish an adaptive stress response, including indole and tryptophanase, which can be regarded as signal molecules. The overexpression of tryptophanase may indicate that the protein is involved in the cell adaptation process of *K. oxytoca*, which can reduce or even offset the negative effects of nZVI. In addition, the activities of some common intracellular antioxidants, such as glutathione reductase (GR), superoxide dismutase (SOD) and catalase (CAT), also change in response to elevated intracellular ROS levels. For example, catalase, encoded by the katB gene, is involved in cellular oxidative stress detoxification. Upregulation of katB occurred after exposure of *p.stutzeri* to nZVI. This phenomenon indicates involvement of katB in H_2_O_2_-induced oxidative stress. The mechanism of microbial toxicity when exposed to nZVI was proposed, as shown in Figure 7. The potential mechanisms of toxicity and cellular defense behavior of nZVI particles are summarized in Figure 8.

## 8. Methods to Alleviate the Toxicity of Nano-Zero-Valent Iron

As mentioned above, the factors that influence nZVI toxicity are complex. Although most toxicity studies have been conducted with bare nZVI, nZVI particles used for in situ remediation are usually surface-stabilized, and the type of stabilizer used not only increases the mobility of nZVI but also attenuates the toxicity of nZVI. Currently, surface modification is used to improve the stability of nZVI due to the limited nature of nZVI itself. At the same time, the only artificially controllable factor to reduce the toxicity of nZVI is the modification method of nZVI. The selection and preparation of modified materials have an impact on the toxicity of nZVI, and the selection of green synthetic modified coating materials can effectively mitigate the toxicity of nZVI. Various types of surface modifiers/stabilizers, such as surfactants, polymeric organics and polyelectrolytes, are used as coating materials to reduce agglomeration/rusting and improve the flowability of nZVI. These stabilizers can provide stability to nanoparticles by applying stabilizing forces (e.g., electrostatic repulsion) and spatial or even electrospatial stabilization between nanoparticles. The electrostatic repulsion (a combined form of electrostatic and spatial repulsion) provided by the large molecular weight polyelectrolyte significantly inhibits the physical contact between nZVI and the bacterial cell wall in the stabilization of nano-zero-valent iron by the spatial repulsion promoted by the negatively charged polymer coating. Thus, it reduces the toxicity of nZVI to microorganisms [42]. In addition, it was shown that the toxicity of nZVI in soil is temporary, and it decreases gradually with oxidative aging of nZVI. The conversion of iron nanoparticles to less toxic or even non-toxic iron oxide leads to a significant reduction in cytotoxicity. The amount of this material is a key factor in toxicity despite the availability of coating material. It has been shown that the increase in the hydrodynamic size of the particles leads to an increase in the biocompatibility of PVA-covered SPION (superparamagnetic iron oxide nanoparticles), which could be the result of a higher polymer/iron mass ratio. This is why it is necessary to control the amount or dosage of stabilizers or coating materials to avoid adverse effects on toxicity.

## 9. Conclusions and Perspectives

As an emerging material for environmental remediation, the negative effects of nZVI on the environment need to be focused on. However, the toxic effects and influencing factors of nZVI in the environment are complex, and the current characterization methods are very limited, so our knowledge of nZVI is still in the initial stage. Studies on the toxic effects of nZVI are just beginning at this stage, and not much research has been conducted in real environments. There are many challenges for future toxicological studies on nZVI on soil microorganisms.

(1) The factors affecting the toxicity of nZVI to soil microorganisms are complex and are influenced by the nature of nZVI itself, whether it is modified, the amount used, and even in practical applications, the soil properties and the linkage with other substances in the environment. In addition, the result of the interaction between nZVI and other substances in the soil in practical application may enhance the toxicity of nZVI or weaken it, and strengthening the research in this aspect is beneficial to determine the performance of nZVI in the actual environment.
(2) The mechanisms of nZVI toxicity are currently agreed upon by academics as morphological or functional damage to microbial cell membranes, oxidative damage, release of iron ions and genetic damage. The first three are toxicological studies at the cellular level, while gene damage is a study at the molecular level and is still in the research stage. Although there are many hypotheses about the mechanism of toxicity, the exact mechanism is still unclear, and the existence of other mechanisms needs to be further investigated, taking into account environmental factors, test organisms and the properties of nZVI itself.
(3) The defenses of microbial cells in soil against nZVI toxicity are currently more clearly elucidated at the cellular level, but studies at the genetic level are still lacking. Meanwhile, the commonalities and characteristics of cellular defense behaviors need to be further investigated and summarized.
(4) Toxicity studies on the temporal effects of nZVI need to be strengthened. nZVI toxicity may be a long-term and chronic process after injection into the environment, and under certain conditions, the short-term toxic effects may not be obvious, so there is a need to strengthen the long-term and large-scale studies on the toxic effects of nZVI on the ecological environment.

## Data Availability

Not applicable.
